# Application of deep learning-clinical baseline feature fusion model to predict postoperative mortality in elderly patients with hip fracture: a multicenter study

**DOI:** 10.3389/fmed.2026.1784156

**Published:** 2026-04-07

**Authors:** Jiasen Hu, Enli Li, Ya-ping Jin, Ji-kui Wu, Jianguang Ni

**Affiliations:** 1 Department of Orthopaedics, Affiliated Yueqing Hospital of Wenzhou Medical University, Wenzhou, Zhejiang, China; 2 Department of Orthopaedics, The Second Affiliated Hospital and Yuying Children's Hospital of Wenzhou Medical University, Wenzhou, Zhejiang, China

**Keywords:** artificial intelligence, deep learning, fusion model, hip fracture, mortality

## Abstract

**Background:**

A large number of studies have focused on building different models to predict postoperative mortality in elderly patients with hip fractures, including sarcopenia risk factor models or deep learning models. However, relying on deep learning models alone may not fully capture the key factors that affect patient outcomes, so it may be a more accurate model to construct predictive models combining clinical baseline features.

**Methods:**

A deep learning model (Densenet161) and a deep learning-clinical baseline feature fusion model (LightGBM) were constructed using 221 patients from Institution 1 as the internal training set and 113 from Institution 2 as the external validation set, respectively. We selected the skeletal muscle tissue image of the 12th thoracic vertebral cross section in the chest CT (computerized tomography) scan as the input data of the Densenet161 model. The model’s predictive performance was evaluated using AUC (area under the curve), sensitivity, specificity, and F1 scores.

**Results:**

The Densenet161 model has an average performance in predicting 1-year postoperative mortality in elderly patients with hip fractures, with an AUC of 0.723 and an F1 score of 0.421 on the external validation set. Compared with Densenet161 model, the predictive performance of LightGBM fusion model has been greatly improved, with AUC of 0.815 and F1 score of 0.819 on the external validation set.

**Conclusion:**

Combining the image features extracted by the deep learning model with the patient’s clinical baseline characteristics, the LightGBM fusion model can better predict the 1-year mortality of elderly hip fracture patients than relying on a single deep learning model.

## Introduction

The rapid social economy and medical technology development have dramatically improved human life expectancy. With the increase of age, the skeletal system of the elderly is gradually degraded, bone density is reduced, bone strength is weakened, and accidents such as falls in daily life make the incidence of hip fracture in the elderly population remain high (1–3). The cumulative 1-year mortality rate for hip fractures in older adults is 33%, and the 1-year mortality rate increases significantly by 2% per year (4). Due to the high mortality rate of hip fractures in older people, hip fracture is also known as the last fracture in the life of the elderly. Therefore, more and more studies have been conducted to find the risk factors affecting the prognosis of elderly patients with hip fractures through various research methods so that timely intervention can be carried out for patients (5).

Sarcopenia, defined as a syndrome of sustained loss of skeletal muscle mass, strength, and function, is a significant health problem in the elderly and in patients with poor functioning (6). Sarcopenia can be a critical prognostic factor in elderly or poorly functioning patients undergoing surgical treatment, significantly affecting the surgical outcome and recovery process (7). Similarly, in elderly patients with hip fractures, sarcopenia has also been shown to be an essential prognoses factor, as it directly affects the patient’s strength, balance, and recovery potential (8). In recent years, evaluation methods based on medical imaging technology, especially the use of chest CT (computerized tomography) scans, have provided a new way to screen and diagnose sarcopenia. Tan et al. (9) found that sarcopenia patients can be effectively identified by measuring the skeletal muscle index (SMI) at the 12th thoracic vertebral plane on chest CT. This method is simple and feasible and has high sensitivity and specificity. More importantly, SMI is not only associated with sarcopenia but also with osteoporosis, fragility fracture, and fracture prognosis (10, 11). In addition, chest CT is a common perioperative examination for elderly patients with hip fractures, employed to assess their cardiopulmonary function, pulmonary inflammation, pleural effusion and other clinical conditions. In addition, chest CT is an essential perioperative examination for elderly patients with hip fractures to assess the condition of the patient’s heart and respiratory system.

In recent years, AI (artificial intelligence) technology has been a spurt of development in the medical field, especially in image recognition and classification (12–14). In medical image recognition and classification, artificial intelligence technology can automatically extract the key features of the image by learning a large amount of medical image data and carrying out accurate classification and recognition. For example, based on images of muscle tissue using CT technology, AI models can effectively identify potential patients with osteoporosis through deep learning and image processing algorithms (15, 16). Applying this technology improves the accuracy and efficiency of diagnosis and provides patients with earlier intervention and treatment opportunities. In addition to the field of image recognition classification, AI technology also shows great potential in medical prediction models. Li et al. (5) built a machine learning model to predict the prognosis of patients with hip fractures based on clinical baseline characteristics such as age and BMI (body mass index).

In summary, this study initially attempted to construct a deep learning model (Densenet161) based on the skeletal muscle images of the 12th thoracic vertebral section of CT to predict the one-year postoperative mortality of elderly patients with hip fractures. With deep learning models, we can automatically extract critical features from skeletal muscle images and make accurate predictions based on them. However, in some specific tasks, relying on deep learning models alone may not make the most of all available information. In particular, when it comes to predicting the prognosis of patients, in addition to skeletal muscle images, the patient’s clinical baseline characteristics (such as age, gender, BMI, etc.) also play an essential role. Therefore, this study proposes constructing a fusion model (LightGBM) combining deep learning features and clinical baseline features to predict 1-year postoperative mortality in elderly patients with hip fractures. By fusing these two types of features, we can gain a more comprehensive understanding of a patient’s health status and potential risks and, thus, more accurately predict a patient’s postoperative mortality. Ultimately, through this study, our team expects to provide a better AI model to predict postoperative mortality in elderly patients with hip fractures. This model will provide an essential reference for clinicians to help them develop more rational treatment and rehabilitation plans to improve patient outcomes and quality of life. At the same time, this research will also provide new ideas and methods for applying deep learning in the medical field and promote the development of medical intelligence.

## Materials and methods

### Study design

This study conducted a retrospective analysis of clinical data collected from patients at the Second Affiliated Hospital of Wenzhou Medical University (Institution 1) and Affiliated Yueqing Hospital of Wenzhou Medical University (Institution 2). The data covered the time frame from January 1, 2023 to April 30, 2023. Before conducting the study, the institutional review boards at participating institutions reviewed and approved the retrospective multicohort study design. Additionally, they granted a waiver for obtaining written informed consent from the patients involved.

**Figure 1:**
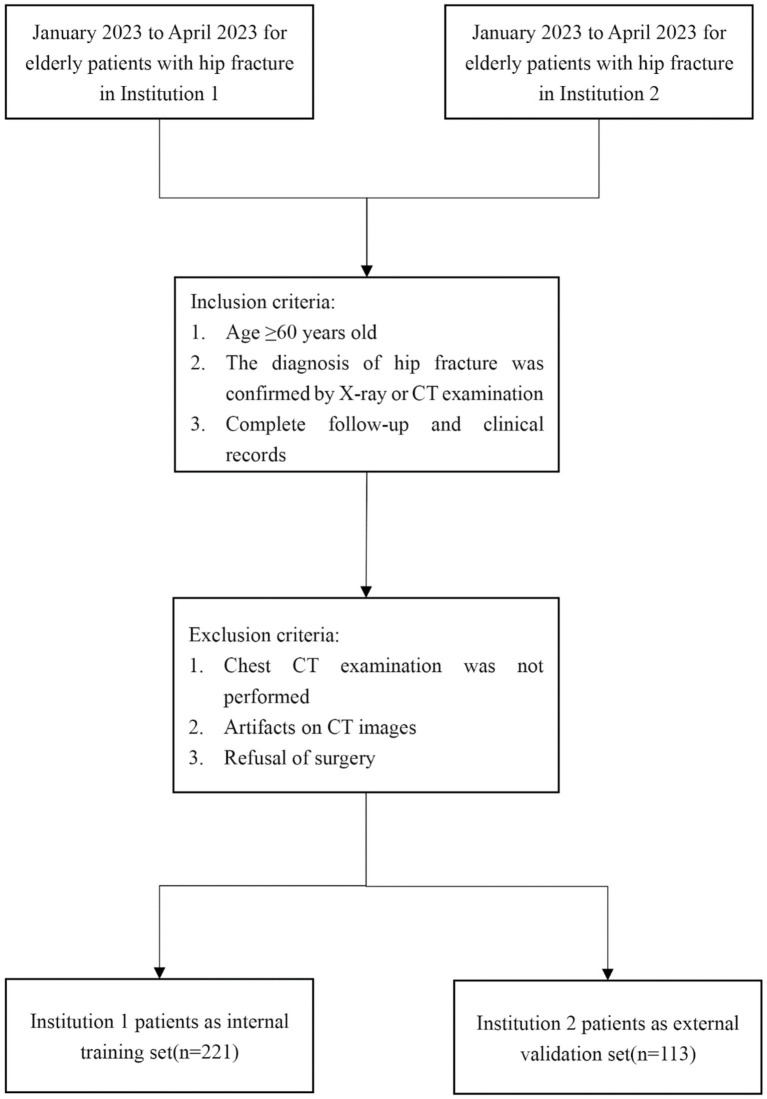
The recruitment process of patients in this study. CT, computed tomography.

**Figure 2:**
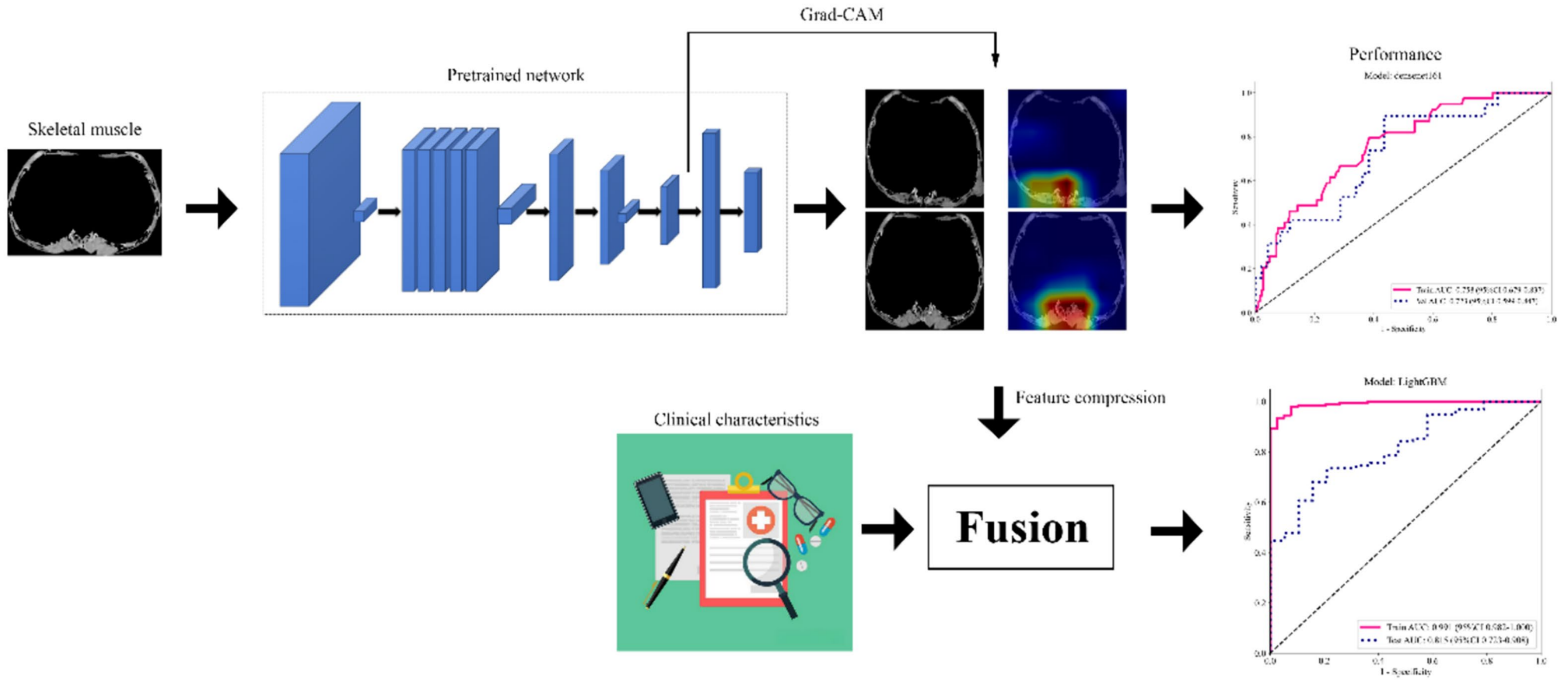
The research process of this study. Grad-CAM, gradient-weighted class activation mapping.

For the present study, we retrospectively enrolled a total of 334 patients from two participating institutions, namely Institution 1 (*n* = 221) and Institution 2 (*n* = 113). The inclusion criteria were as follows: (1) age ≥60 years [the definition of older adults in this study, referencing the cohort study on geriatric hip fracture by Chuang et al. (17)], (2) The diagnosis of hip fracture was confirmed by X-ray or CT examination, and (3) Complete follow-up and clinical records. On the other hand, the exclusion criteria were: (1) Chest CT examination was not performed, (2) Artifacts on CT images, and (3) Refusal of surgery. Among the participating institutions, Institution 1 enrolled 221 patients, while Institution 2 included 113 patients. The patient recruitment process for this study is illustrated in Figure 1. Moreover, Figure 2 shows the specific ideas of this study.

Unenhanced chest CT images with a slice thickness of 5 mm from Institution 1 and Institution 2 were retrieved from the picture archiving and communication systems (PACS). Furthermore, all CT images were standardized by resampling them to a uniform voxel spacing of 1 * 1 * 1 mm^3^. Concurrently, all images’ window levels and widths were adjusted to 40 and 300, respectively, to ensure consistency in image visualization. Institution 1 and Institution 2 followed similar clinical guidelines and data collection standards, ensuring minimal differences in data quality.

### Clinical baseline variables and outcome definition

Based on clinical practice and published literature on hip fracture prognosis, a total of 7 perioperative clinical baseline variables were selected as potential predictive factors for the fusion model in this study, with no additional clinical variables included in the subsequent feature screening and model construction. All these 7 variables were collected and measured during the perioperative period, specifically within 24 h after patient admission to the orthopaedic ward and prior to the performance of hip fracture surgery, based on the first comprehensive clinical assessment data. The operational definitions of the 7 perioperative clinical baseline variables were standardized as follows: (1) Age: the actual chronological age of patients in full years at the time of admission; (2) Gender: the biological gender (male/female) of patients as recorded in the electronic medical record (EMR); (3) Body mass index (BMI): calculated using the formula weight (kg)/height^2^ (m^2^), with weight and height measured by trained nursing staff under standardized conditions (patients without shoes and in light clothing); (4) American Society of Anesthesiologists (ASA) score: evaluated by an attending anesthesiologist in accordance with the ASA physical status classification system during preoperative anesthetic assessment; (5) Fracture type: categorized as intertrochanteric fracture or femoral neck fracture, confirmed by the chief orthopaedic surgeon based on preoperative X-ray and CT imaging results; (6) Anesthesia method: classified as general anesthesia or other anesthesia methods, determined and recorded by the attending anesthesiologist; (7) Surgical procedure: including Proximal Femoral Nail Antirotation (PFNA), hip arthroplasty, and cannulated screw fixation, confirmed by the chief surgeon and documented in the official surgical record.

The primary outcome of this study was defined as 1-year all-cause mortality after hip fracture surgery, referring to all-cause death within 12 months post-surgery with the operation date as the starting point and the date of death or the 12th month post-surgery as the endpoint for surviving patients. This endpoint was selected because it holds the most clinical significance for elderly hip fracture patients and is consistent with the primary outcome measures in most relevant studies. The ascertainment of death date adopted a multi-source verification approach to ensure the accuracy of survival status: in-hospital death data were directly extracted from the electronic medical record (EMR) systems of the two participating institutions, while out-of-hospital death information was obtained via standardized telephone follow-up conducted by dedicated orthopaedic follow-up nurses. A standardized follow-up protocol was implemented for all included patients.

### Densenet161 model construction

A professional clinician used 3D slicer software (version 5.2.2) to sketch the skeletal muscle tissue of the 12th thoracic vertebral cross-section, and another senior clinician checked the skeletal muscle tissue. All clinicians are blind to patient information when mapping and checking skeletal muscle tissue.

The sketched skeletal muscle tissue images were put into the Densenet161 deep learning model to predict 1-year mortality in elderly patients after hip fracture surgery. During model construction, the CT image data of patients from Institution 1 served as the internal training set, while the CT image data from Institution 2 functioned as the external validation set. Before training, the input CT images were resized to 224 × 224 pixels to match the requirements of the Densenet161 model. An SGD (stochastic gradient descent) optimizer was employed to update the model’s parameters, with an initial learning rate of 0.01 was dynamically adjusted using the cosine annealing algorithm (this method cyclically adjusts the learning rate, allowing the model to escape local minima and converge more effectively, which can improve generalization). The training process encompassed 200 epochs, with a constant batch size of 32 images per iteration. Besides, Densenet161 includes batch normalization layers that normalize the inputs of each layer. This helps to stabilize the learning process and reduce overfitting by ensuring that each layer receives inputs with similar distributions. Additionally, we used data from a second institution as an external validation set. This external validation serves as a crucial step in assessing the model’s ability to generalize beyond the training data and provides an objective evaluation of its performance in a different clinical setting.

We utilized the Grad-CAM (Gradient-weighted Class Activation Mapping) technique (18) to visualize activations in the final convolutional layer of the Densenet161 model. Grad-CAM provides valuable insights into the critical elements deep learning models consider when making decisions, enhancing our understanding of image information identification. Additionally, we extracted and compressed features from the last convolutional layer of the Densenet161 model, facilitating subsequent fusion construction. Specifically, features were extracted from the last convolutional layer of the pre-trained Densenet161 model (prior to the fully connected layer), and for the CT images resized to 224 × 224 pixels in accordance with the model input requirements, this layer generated a high-dimensional original deep learning feature set with a dimension of 16,383. Dimensionality reduction of these 16,383 features was then performed using Principal Component Analysis (PCA), a gold-standard feature compression method in deep learning. The number of principal components was selected based on the cumulative variance explanation rate criterion (>95%), which ensured that the retained features captured the vast majority of information from the original high-dimensional feature set. Ultimately, 32 principal components were retained as the compressed deep learning features, which were subsequently integrated with clinical candidate features to form the fused feature set for fusion model construction.

### Fusion model construction

A fusion model was constructed to predict 1-year postoperative mortality in elderly patients with hip fractures by fusing compressed deep learning features (*n* = 32) with patient clinical baseline features (*n* = 7). LightGBM and SVM were used as fusion model algorithms, which were similar to the construction of Densenet161 model. In this study, the SVM and LightGBM fusion models utilized the exact same set of clinical baseline features and deep learning compressed features, ensuring the fairness of inter-model comparisons and the reliability of the study findings. Data from institution 1 were used as the internal training set, and data from institution 2 were used as the external validation set. To assess the risk of overfitting during model development, 5-fold cross-validation was conducted as the internal validation method on the internal training set (Institution 1). The training set was randomly and stratifiably split into 5 mutually exclusive subsets with consistent 1-year mortality distribution; the model was trained on 4 subsets and validated on the 1 remaining subset, with this process repeated 5 times for full cross-validation. All training set performance metrics reported in this study represent the average values of the 5-fold cross-validation results.

The fused features undergo *Z*-score normalization to standardize their scale. Initially, a screening process is applied using the Spearman rank correlation coefficient, retaining only one feature from any pair of features with a correlation coefficient greater than 0.9. This step aims to reduce redundancy in the feature set. Finally, the Least Absolute Shrinkage and Selection Operator (LASSO) algorithm was utilized to identify the most informative and predictive final feature subset, with the algorithm implemented on the internal training set (Institution 1) via 5-fold cross-validation to select the optimal lambda value based on the minimum mean squared error criterion. A total of 14 effective predictors were retained in the final model, including 12 compressed deep learning features and 2 perioperative clinical baseline features. For the Fusion model, we employed the LASSO algorithm during feature selection. LASSO not only identifies the most predictive features but also applies regularization to shrink less important feature coefficients toward zero. This regularization effect helps prevent the model from fitting too closely to the noise in the training data.

### Statistical analysis

The Shapiro–Wilk test was applied to assess clinical baseline characteristic data distribution. The Mann–Whitney *U* test is used for continuous variables that are not normally distributed, and the Pearson Chi-square test is used for categorical variables. Continuous variables are represented by the number of quarterbacks, and categorical variables are represented by percentages. Performance metrics, including sensitivity, specificity, F1-score (19), calibration curve and the AUC (area under the curve), were used to evaluate the different models. Data was processed using SPSS (version 26.0; SPSS Inc., Chicago, IL, USA) and Python (version 3.9.7).

## Results

### Clinical baseline characteristics and performance of the clinical prediction model

Three hundred thirty-four elderly patients with hip fractures were enrolled in this study: 221 were enrolled in Institution 1, and 113 were enrolled in Institution 2. These patients were divided into a death group and a survival group according to their survival 1 year after surgery. The number of patients in the death and survival groups at Institution 1 was 39 and 182, respectively, and the mortality rate 1 year after surgery was approximately 17%. The number of patients in the death and survival groups at Institution 2 was 19 and 94, respectively, and the mortality rate 1 year after surgery was about 16%. In addition, the age and ASA (American Society of Anesthesiologists) scores of Institution 1 and Institution 2 were significantly different in the death and survival groups (*p* < 0.05). There were no significant differences in other clinical baseline characteristics (details are shown in Table 1). ASA score and type of anesthesia were significantly different between the two institutions, and there were no significant differences in age, BMI, sex, fracture type, and surgical procedure (see Table 2 for details). Furthermore, a logistic regression model was developed using the aforementioned clinical baseline variables to predict 1-year all-cause mortality among elderly patients with hip fractures. The model’s predictive performance was assessed in both cohorts, yielding area under the receiver operating characteristic curve (AUC) values of 0.789 for Institution 1 and 0.774 for Institution 2 (Supplementary Figure S1).

**Table 1:** Clinical baseline characteristics of the participating populations at both institutions.

Variable	Institution 1			Institution 2		
	Death group (*n* = 39)	Survival group (*n* = 182)	*p-*value	Death group (*n* = 19)	Survival group (*n* = 94)	*p*-value
Age, (years)	88 (82–91)	78 (70–85)	<0.001	84 (82–88)	75 (68–84)	<0.001
BMI, (kg/m^2^)	20.51 (18.67–23.44)	21.85 (19.61–24.45)	0.220	21.80 (19.47–25.71)	22.55 (20.45–24.54)	0.735
Gender, *n* (%)			0.149			0.104
Female	24 (61.5)	133 (73.1)		9 (47.4)	63 (67.0)	
Male	15 (38.5)	49 (26.9)		10 (52.6)	31 (33.0)	
ASA score, *n* (%)			0.002			0.015
I or II	19 (48.7)	135 (74.2)		14 (73.7)	87 (92.6)	
III or above	20 (51.3)	47 (25.8)		5 (26.3)	7 (7.4)	
Anesthesia, *n* (%)			0.834			0.430
General anesthesia	16 (41.0)	78 (42.9)		0 (0.0)	3 (3.2)	
Other anesthesia method	23 (59.0)	104 (57.1)		19 (100)	91 (96.8)	
Fracture type, *n* (%)			0.101			0.189
Intertrochanteric fracture	21 (53.8)	72 (39.6)		11 (57.9)	39 (41.5)	
Femoral neck fracture	18 (46.2)	110 (60.4)		8 (42.1)	55 (58.5)	
Surgical procedure, *n* (%)			0.245			0.249
PFNA	21 (53.8)	72 (39.6)		11 (57.9)	39 (41.5)	
Hip arthroplasty	16 (41.0)	94 (51.6)		8 (42.1)	47 (50.0)	
Cannulated screws	2 (5.1)	16 (8.8)		0 (0.0)	8 (8.5)	

BMI, body mass index; ASA, American Society of Anesthesiologists; PFNA, proximal femoral nail antirotation.

**Table 2:** Clinical baseline characteristics at the two institutions.

Variable	Institution 1 (*n* = 221)	Institution 2 (*n* = 113)	*p*-value
Age, (years)	72 (66–80)	69 (63–78)	0.055
BMI, (kg/m^2^)	21.36 (19.51–24.42)	22.48 (20.24–24.77)	0.084
Gender, *n* (%)			0.173
Female	157 (71.0)	72 (63.7)	
Male	64 (29.0)	41 (36.3)	
ASA score, *n* (%)			<0.001
I or II	154 (69.7)	101 (89.4)	
III or above	67 (30.3)	12 (10.6)	
Anesthesia, *n* (%)			<0.001
General anesthesia	94 (42.5)	3 (2.7)	
Other anesthesia method	127 (57.5)	110 (97.3)	
Fracture type, *n* (%)			0.705
Intertrochanteric fracture	93 (42.1)	50 (44.2)	
Femoral neck fracture	128 (57.9)	63 (55.8)	
Surgical procedure, *n* (%)			0.901
PFNA	93 (42.1)	50 (44.2)	
Hip arthroplasty	110 (49.8)	55 (48.7)	
Cannulated screws	18 (8.1)	8 (7.1)	

BMI, body mass index; ASA, American Society of Anesthesiologists; PFNA, proximal femoral nail antirotation.

### Performance of the Densenet161 model

**Figure 3:**
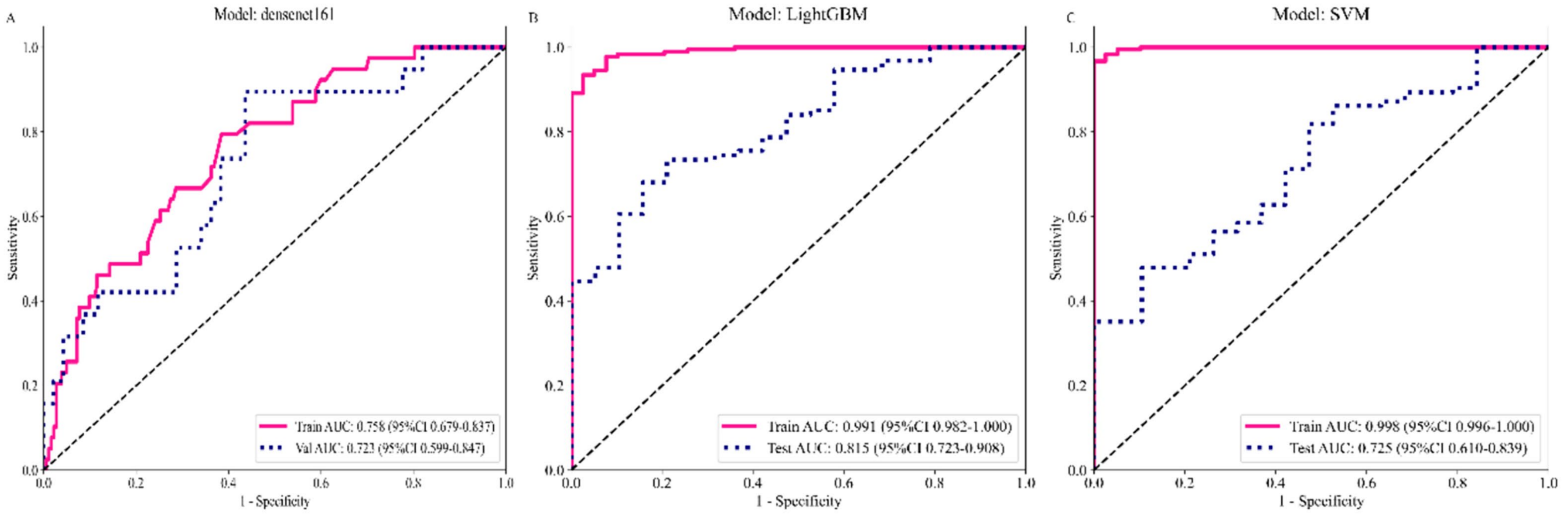
Receiver operating characteristic curves of deep learning models: **(A)** Densenet161, **(B)** LightGBM, and **(C)** SVM in train and validation sets.

**Figure 4:**
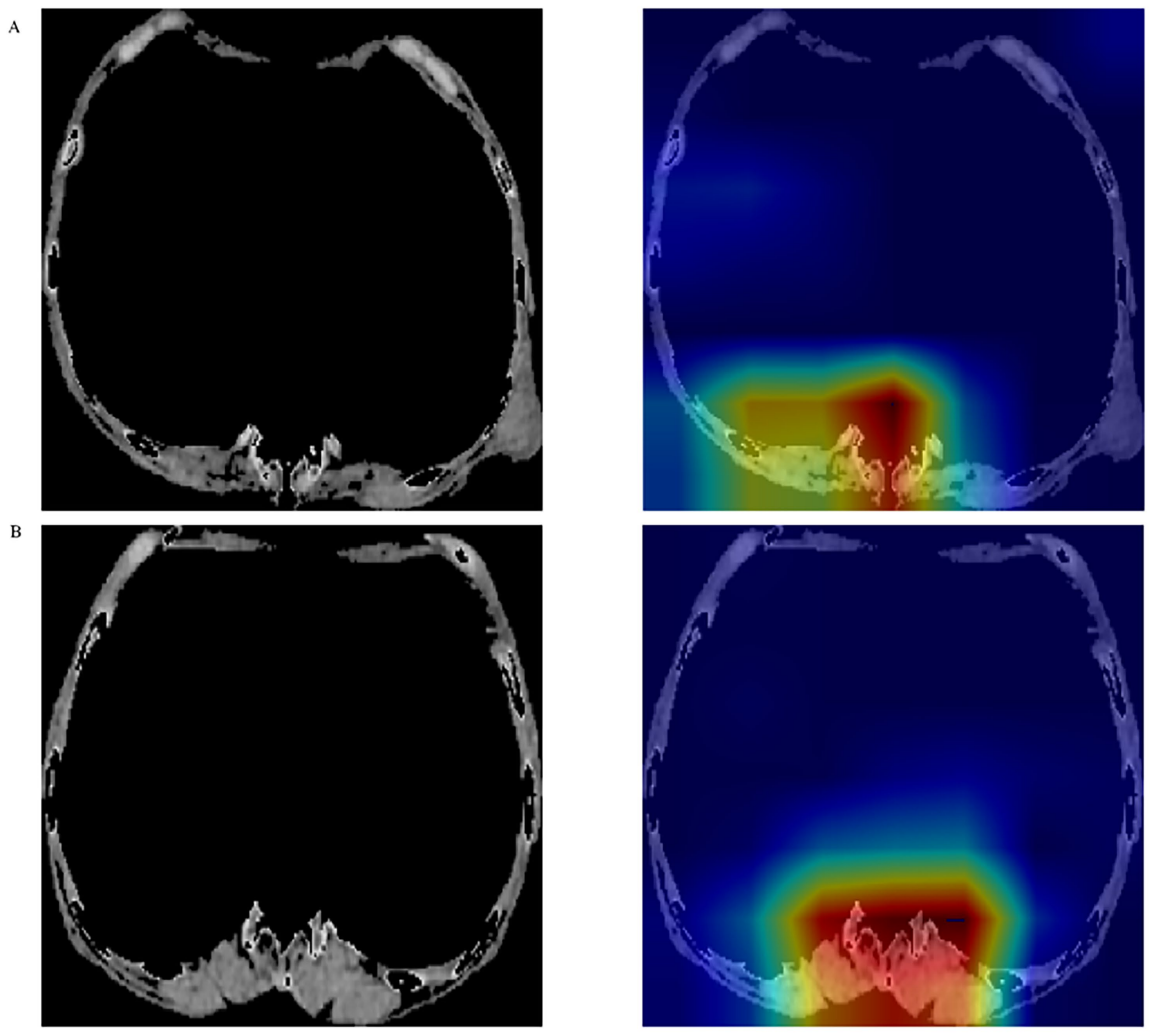
The focus area of the Densenet161 deep learning model in the CT images of **(A)** Institution 1 and **(B)** Institution 2. CT, computed tomography.

The Densenet161 algorithm model predicted 1-year mortality after hip fracture in elderly patients. The prediction performance of the Densenet161 model in both the internal training and external validation sets was average, with AUC values of 0.758 and 0.723 and F1 scores of 0.418 and 0.421, respectively (Table 3 and Figure 3A). However, based on the Grad-CAM algorithm, the last convolutional layer of the Densenet161 model was visualized. This study found that the focus area of the Densenet161 model was musculoskeletal tissue in both the data sets of Institution 1 and Institution 2, but not other regions (Figure 4). In addition, this study extracted 16,383 deep learning features from the last convolutional layer of the Densenet161 model and then compressed these features to get 32 deep learning features.

**Table 3:** Predictive performance of different models.

Model	DataSet	AUC (95% CI)	Sensitivity	Specificity	F1 score
Densenet161	Train	0.758 (0.679–0.837)	0.718	0.632	0.418
	Validation	0.723 (0.599–0.848)	0.842	0.564	0.421
LightGBM	Train	0.991 (0.982–0.999)	0.929	0.974	0.961
	Validation	0.815 (0.723–0.908)	0.723	0.789	0.819
SVM	Train	0.998 (0.996–0.890)	0.967	1.000	0.983
	Validation	0.725 (0.610–0.839)	0.479	0.895	0.638

AUC, area under curve.

### Performance of the fusion model

In this study, LightGBM and SVM algorithms were used to construct fusion models to predict the 1-year mortality of elderly patients with hip fracture. Compared with the Densenet161 model, the prediction performance of LightGBM and SVM fusion model was significantly improved. In addition, the LightGBM model outperforms the SVM model in the external validation set. The AUC and F1 scores of the LightGBM fusion model were 0.991 and 0.961 for the internal training set, and 0.815 and 0.819 for the external validation set, respectively (Table 3 and Figures 3B,C). Supplementary Figure S2 shows the importance ratio of different features in the LightGBM fusion model. Age and ASA score play an essential role in the LightGBM fusion model, while gender does not play a significant role. Furthermore, as illustrated in Supplementary Figure S3, the LightGBM fusion model shows acceptable calibration in both the internal training set and the external validation set.

## Discussion

This study used patient data from Institution 1 as the internal training set and data from Institution 2 as the external validation set. The Densenet161 deep learning model, SVM and LightGBM fusion model were constructed to predict the 1-year postoperative mortality of elderly patients with hip fractures.

As one of the deep learning models of convolutional neural networks, Densenet161 has strong feature extraction ability and complex data processing ability (20). This study, Densenet161 was used to extract critical information from skeletal muscle images of the 12th thoracic vertebral cross-section on CT. By training this model, we expect it to be able to predict mortality 1 year after surgery in elderly patients with hip fractures. The densenet model has achieved good performance in image recognition and classification, such as identifying myocardial infarction patients based on ECG and diagnosing and classifying hip fractures (21, 22). The Densenet161 model visualizes the areas of focus with Grad-CAM technology. As expected, the Densenet161 model focuses on skeletal muscle tissue but not other areas. However, the prediction performance of the Densenet161 model in this study is not very good; its AUC in the external verification set is only 0.723, and the F1 score is only 0.421. Similarly, the predictive models constructed based on clinical variables also perform poorly. Therefore, our team realized that relying solely on image features or clinical variable features might not be sufficient to fully predict the one-year mortality rate of elderly patients with hip fractures after surgery.

We propose a fusion model (LightGBM and SVM) combining deep learning and clinical baseline features. In this study, SVM performed worse than LightGBM on the external validation set, specifically its AUC and F1 scores were lower than the LightGBM model. LightGBM is a highly efficient and highly performing gradient lifting framework used in various medical applications, such as predicting pregnancy outcomes and emergency department trauma patient mortality (23, 24). In this study, the LightGBM model was used to fuse image features extracted from the Desenet161 model with clinical baseline features of patients. This fusion model aims to improve the accuracy of predictions by considering both imaging and clinical information. However, we acknowledge that the overfitting risk of the fusion model requires a comprehensive assessment considering both the limited number of outcome events and the ratio of mortality events to effective predictors. The total number of 1-year all-cause mortality events in our multicenter cohort was 58 (39 from Institution 1 and 19 from Institution 2), corresponding to a ratio of outcome events to the 14 final effective predictors of approximately 4.14:1. This imbalance in the event-to-predictor ratio may elevate the potential risk of model overfitting. To mitigate this issue, multiple rigorous strategies were implemented during model development: stratified 5-fold cross-validation was conducted on the internal training set to optimize hyperparameters and avoid overfitting to individual data subsets; an independent external validation set from a different institution was used to assess model generalizability—the gold standard for validating clinical prediction models; LASSO regularization was applied to shrink coefficients of non-informative features to zero, reducing predictor numbers and avoiding overfitting caused by excessive feature dimensionality; and *Z*-score normalization was performed on fused features to eliminate scale differences and stabilize model training. These strategies collectively enhanced the model’s robustness and mitigated the practical impact of potential overfitting.

Notably, the LightGBM fusion model exhibited a marked improvement in predictive performance compared with the Densenet161 model. The AUC of the LightGBM fusion model external validation set is 0.815, and the F1 score is 0.819. In addition, Supplementary Figure S1 shows the importance of different features of the LightGBM fusion model in predicting postoperative outcomes in elderly patients with hip fractures. From this visualization, it is clear that in the LightGBM fusion model, age and ASA score are two significant features in the model’s prediction. The feature importance analysis in our study highlights age and ASA score as the most predictive features for postoperative mortality risk. While machine learning feature importance reflects the contribution of these features to the model’s predictions and does not establish causation, previous research has demonstrated causal relationships between age and ASA score and postoperative mortality in elderly hip fracture patients. A large number of previous studies have shown that age and ASA score are important risk factors for postoperative mortality in elderly patients with hip fractures (25, 26). The body’s physiological functions gradually decline with age, and the immune system weakens, making elderly patients more vulnerable to infections and other complications (27). At the same time, the ASA score reflects the patient’s general health status and surgical risk; the higher the score, the more health problems the patient has (28). Together, these factors increase the risk of postoperative death in older patients with hip fractures.

Gender is widely recognized as a potential prognostic factor for postoperative mortality in elderly patients with hip fracture in existing published literature, and it is frequently incorporated into clinical predictive models for this population (29–31). However, in the present study, gender was not retained in the final LightGBM fusion model, a result that stemmed from an objective, data-driven feature selection process. The inconsistency between our results and previous literature regarding the prognostic role of gender may be attributed to several cohort-specific and analytical factors. First, the study population was composed of elderly hip fracture patients from a specific regional area in eastern China, and the demographic and clinical characteristics of this cohort (e.g., gender-related differences in pre-fracture functional status, comorbidity profiles) may differ from those of Western or other Asian populations in previous studies, potentially weakening the predictive value of gender. Second, the inclusion of ASA score—a strong, comprehensive indicator of general health status and surgical risk—in the final model may have masked the potential prognostic effect of gender, as ASA score integrates multiple gender-related health covariates that could independently influence postoperative mortality. Thirdly, the features extracted from the imaging data may implicitly reflect physiological characteristics related to gender, which may reduce the significant influence of the gender variable during the model selection process.

The interpretability of the LightGBM fusion model represents a crucial advantage for clinical applications. By providing transparent and understandable explanations of its predictions, the model empowers clinicians to make more informed decisions. Clinicians can rely on the feature importance to identify the key factors driving the model’s predictions for each patient. This information enhances their ability to conduct personalized risk assessments, implement targeted interventions, and optimize treatment plans. For instance, if the model highlights a patient’s high ASA score as a major risk factor, clinicians can focus on preoperative optimization of the patient’s medical conditions to reduce postoperative complications. The model’s interpretability thus bridges the gap between advanced machine learning techniques and practical clinical decision-making, making it a valuable tool for improving patient care.

The selection of age and ASA score as key features in the LightGBM fusion model reflects our goal to develop a tool that is not only predictive but also practical for widespread clinical use. These features were chosen for their universal availability and routine collection in preoperative assessments, which facilitates the model’s integration into diverse healthcare settings. This approach ensures that the model can be applied consistently across different institutions, regardless of resource limitations or variations in clinical practices. While other clinical features were considered, their exclusion was driven by the need to balance predictive performance with clinical practicality. By focusing on features that are easily obtainable, we have created a model that can be adopted more readily, thereby enhancing its potential impact on patient care. This approach aligns with the broader goal of developing predictive tools that are both effective and accessible, ensuring they can be used to improve outcomes for elderly hip fracture patients in various clinical contexts.

The integration of the LightGBM fusion model into clinical practice involves addressing several practical barriers, particularly regarding technical infrastructure and clinician training. Technically, the model requires access to high-quality chest CT imaging equipment and a reliable PACS for data storage. While the LightGBM model can be deployed on standard hospital computers, the Densenet161 component necessitates more substantial computational resources, ideally with GPUs, for efficient training. This may pose challenges for hospitals with limited technological capacity. However, these hospitals can collaborate with research institutions or medical technology companies to access the necessary computational infrastructure. Additionally, cloud-based solutions can be leveraged for both data storage and computationally intensive training processes. Clinician training is another critical aspect. By focusing on how the model’s predictions can enhance patient care, clinicians can effectively utilize the model to improve outcomes for elderly patients with hip fractures.

The LightGBM fusion model methodology exhibits considerable adaptability for predicting outcomes in other medical or surgical contexts. By integrating imaging features with clinical baseline characteristics within a gradient boosting framework, this approach offers a flexible foundation that can be customized to various pathologies. For instance, in predicting postoperative recovery for orthopedic surgeries or cardiovascular interventions, the model could be modified to incorporate imaging biomarkers and clinical features relevant to these specific contexts. To generalize the model to other pathologies, several key modifications would be necessary. First, the feature selection process would need to be re-conducted to identify the most predictive imaging and clinical features for the new outcome of interest. This might involve incorporating different imaging modalities and clinical indicators depending on the specific medical context. Second, the model would require retraining on a dataset specific to the target pathology, with appropriate adjustments to hyperparameters and validation strategies. External validation across multiple institutions would also be essential to ensure the model’s generalizability. Furthermore, collaboration with clinicians specializing in the relevant field would be vital to ensure the model’s clinical relevance and interpretability.

Although this study has achieved some meaningful results, it has some limitations. First of all, as this study adopts a retrospective cohort study design, it will inevitably be affected by some biases, such as differences in ASA scores and anesthesia methods between the two institutions, which may cause specific interference with the research results. Second, critical preoperative comorbidities and concomitant injuries (advanced malignant tumors, hip fracture-associated traumatic brain injuries, and other life-threatening underlying diseases) were not included in our exclusion criteria. These well-recognized confounding variables influence the 1-year postoperative mortality of elderly hip fracture patients, and their omission impaired the integrity of the model feature set, potentially compromising predictive accuracy. Third, specific comorbidities (e.g., hypertension, diabetes mellitus, and coronary heart disease) along with the number and severity of such conditions were not incorporated into the model analysis. This omission was due to our focus on selecting quantifiable clinical baseline features with high multicenter consistency (e.g., age and ASA score) during the initial feature screening. Comorbidities are well-recognized important potential predictors of postoperative mortality in elderly hip fracture patients, and their exclusion may limit the comprehensiveness of the model’s feature set. In subsequent studies, we will integrate detailed information on various comorbidities and their control status into the feature set, and perform stratified analysis to further optimize the predictive performance of the model. Besides, the sample size of this study is relatively small, which may limit the wide applicability and reliability of the results, such as the possibility of overfitting the model. Although we used data from multi-center sources to build and validate the model to increase the reliability of the study, the need for more sample size is still an issue that needs attention. Notably, the ratio of 1-year mortality events to effective predictors in the final model is lower than the ideal clinical research criterion, which further increases the potential overfitting risk. Although we used data from multi-center sources to build and validate the model to increase the reliability of the study, the need for more sample size is still an issue that needs attention. In future studies, we will expand the multicenter sample size and increase the number of mortality outcome events to optimize the event-predictor ratio, which is expected to further reduce the overfitting risk and improve the model’s generalizability and robustness. Finally, the LightGBM fusion model shows potential for scalability in larger multicenter studies. Its reliance on commonly collected clinical features and standardized imaging data makes it adaptable to diverse healthcare systems. However, challenges such as variability in data formats and imaging protocols must be addressed. Future studies can implement data standardization, centralized imaging processing, and comprehensive feature selection to ensure consistent data quality and model performance across different institutions. To address these limitations, we recommend that future research should focus on validating the model with larger, multi-center datasets. This would provide a more comprehensive assessment of the model’s generalizability and robustness. Additionally, prospective cohort studies could be valuable in further refining the model and ensuring its reliability across different patient populations and clinical environments.

## Conclusion

Combining the image features extracted by the deep learning model with the patient’s clinical baseline characteristics, the LightGBM fusion model can better predict the 1-year mortality of elderly hip fracture patients than relying on a single deep learning model.

## Data Availability

The raw data supporting the conclusions of this article will be made available by the authors, without undue reservation.

## References

[1] Xia J Li Z Zhao D Hu Y Lu X. Prevalence of frailty among elderly patients with hip fracture in China: protocol for a systematic review and meta-analysis. BMJ Open. (2023) 13:e072623. doi: 10.1136/bmjopen-2023-072623, 37852775 PMC10603504

[2] Cummings SR Melton LJ. Epidemiology and outcomes of osteoporotic fractures. Lancet. (2002) 359:1761–7. doi: 10.1016/s0140-6736(02)08657-9, 12049882

[3] Huang C Chen X Wu D Chen J Wang J Li S . Autophagic damage in senescent bone marrow mesenchymal stromal cells: impact on Piezo1 expression during osteoporosis progression. Int J Biol Macromol. (2025) 330:147928. doi: 10.1016/j.ijbiomac.2025.147928, 41015372

[4] Guzon-Illescas O Perez Fernandez E Crespí Villarias N Quirós Donate FJ Peña M Alonso-Blas C . Mortality after osteoporotic hip fracture: incidence, trends, and associated factors. J Orthop Surg Res. (2019) 14:203. doi: 10.1186/s13018-019-1226-6, 31272470 PMC6610901

[5] Li Y Chen M Lv H Yin P Zhang L Tang P. A novel machine-learning algorithm for predicting mortality risk after hip fracture surgery. Injury. (2021) 52:1487–93. doi: 10.1016/j.injury.2020.12.008, 33386157

[6] Sayer AA Cruz-Jentoft A. Sarcopenia definition, diagnosis and treatment: consensus is growing. Age Ageing. (2022) 51:afac220. doi: 10.1093/ageing/afac220, 36273495 PMC9588427

[7] Fang P Zhou J Xiao X Yang Y Luan S Liang Z . The prognostic value of sarcopenia in oesophageal cancer: a systematic review and meta-analysis. J Cachexia Sarcopenia Muscle. (2023) 14:3–16. doi: 10.1002/jcsm.13126, 36415154 PMC9891912

[8] Inoue T Maeda K Nagano A Shimizu A Ueshima J Murotani K . Undernutrition, sarcopenia, and frailty in fragility hip fracture: advanced strategies for improving clinical outcomes. Nutrients. (2020) 12:3743. doi: 10.3390/nu12123743, 33291800 PMC7762043

[9] Tan L Ji G Bao T Fu H Yang L Yang M. Diagnosing sarcopenia and myosteatosis based on chest computed tomography images in healthy Chinese adults. Insights Imaging. (2021) 12:163. doi: 10.1186/s13244-021-01106-2, 34743259 PMC8572237

[10] Huang CB Lin DD Huang JQ Hu W. Based on CT at the third lumbar spine level, the skeletal muscle index and psoas muscle index can predict osteoporosis. BMC Musculoskelet Disord. (2022) 23:933. doi: 10.1186/s12891-022-05887-5, 36280811 PMC9590212

[11] Iida H Seki T Sakai Y Watanabe T Wakao N Matsui H . Low muscle mass affect hip fracture treatment outcomes in older individuals: a single-institution case-control study. BMC Musculoskelet Disord. (2021) 22:259. doi: 10.1186/s12891-021-04143-6, 33750363 PMC7945055

[12] Chen ZH Lin L Wu CF Li CF Xu RH Sun Y. Artificial intelligence for assisting cancer diagnosis and treatment in the era of precision medicine. Cancer Commun (London, England). (2021) 41:1100–15. doi: 10.1002/cac2.12215, 34613667 PMC8626610

[13] Zhong F Xing J Li X Liu X Fu Z Xiong Z . Artificial intelligence in drug design. Sci China Life Sci. (2018) 61:1191–204. doi: 10.1007/s11427-018-9342-2, 30054833

[14] Huang C Li E Hu J Huang Y Wu Y Wu B . Enabling early identification of malignant vertebral compression fractures through 2.5D convolutional neural network model with CT image analysis. Spine Phila Pa 1976. (2025) 50:1728–36. doi: 10.1097/brs.0000000000005438, 40548499

[15] Huang CB Hu JS Tan K Zhang W Xu TH Yang L. Application of machine learning model to predict osteoporosis based on abdominal computed tomography images of the psoas muscle: a retrospective study. BMC Geriatr. (2022) 22:796. doi: 10.1186/s12877-022-03502-9, 36229793 PMC9563158

[16] Huang C Wu D Wang B Hong C Hu J Yan Z . Application of deep learning model based on unenhanced chest CT for opportunistic screening of osteoporosis: a multicenter retrospective cohort study. Insights Imaging. (2025) 16:10. doi: 10.1186/s13244-024-01817-2, 39792306 PMC11723875

[17] Chuang SH Kuo YJ Huang SW Zhang HW Peng HC Chen YP. Association between long-term exposure to air pollution and the rate of mortality after hip fracture surgery in patients older than 60 years: Nationwide cohort study in Taiwan. JMIR Public Health Surveill. (2024) 10:e46591. doi: 10.2196/46591, 38342504 PMC10985614

[18] Zhang H Ogasawara K. Grad-CAM-based explainable artificial intelligence related to medical text processing. Bioengineering. (2023) 10:1070. doi: 10.3390/bioengineering10091070, 37760173 PMC10525184

[19] DeVries Z Locke E Hoda M Moravek D Phan K Stratton A . Using a national surgical database to predict complications following posterior lumbar surgery and comparing the area under the curve and F1-score for the assessment of prognostic capability. Spine J. (2021) 21:1135–42. doi: 10.1016/j.spinee.2021.02.007, 33601012

[20] Zhou T Ye X Lu H Zheng X Qiu S Liu Y. Dense convolutional network and its application in medical image analysis. Biomed Res Int. (2022) 2022:2384830. doi: 10.1155/2022/2384830, 35509707 PMC9060995

[21] Jahmunah V Ng EYK Tan RS Oh SL Acharya UR. Uncertainty quantification in DenseNet model using myocardial infarction ECG signals. Comput Methods Prog Biomed. (2023) 229:107308. doi: 10.1016/j.cmpb.2022.107308, 36535127

[22] Cha Y Kim JT Park CH Kim JW Lee SY Yoo JI. Artificial intelligence and machine learning on diagnosis and classification of hip fracture: systematic review. J Orthop Surg Res. (2022) 17:520. doi: 10.1186/s13018-022-03408-7, 36456982 PMC9714164

[23] Li L Cui X Yang J Wu X Zhao G. Using feature optimization and LightGBM algorithm to predict the clinical pregnancy outcomes after in vitro fertilization. Front Endocrinol. (2023) 14:1305473. doi: 10.3389/fendo.2023.1305473, 38093967 PMC10716466

[24] Lee S Kang WS Kim DW Seo SH Kim J Jeong ST . An artificial intelligence model for predicting trauma mortality among emergency department patients in South Korea: retrospective cohort study. J Med Internet Res. (2023) 25:e49283. doi: 10.2196/49283, 37642984 PMC10498319

[25] Chen M Du Y Tang W Yu W Li H Zheng S . Risk factors of mortality and second fracture after elderly hip fracture surgery in Shanghai, China. J Bone Miner Metab. (2022) 40:951–9. doi: 10.1007/s00774-022-01358-y, 35939235

[26] Barceló M Torres OH Mascaró J Casademont J. Hip fracture and mortality: study of specific causes of death and risk factors. Arch Osteoporos. (2021) 16:15. doi: 10.1007/s11657-020-00873-7 33452949

[27] Fulop T Larbi A Pawelec G Khalil A Cohen AA Hirokawa K . Immunology of aging: the birth of inflammaging. Clin Rev Allergy Immunol. (2023) 64:109–22. doi: 10.1007/s12016-021-08899-6, 34536213 PMC8449217

[28] Mayhew D Mendonca V Murthy BVS. A review of ASA physical status - historical perspectives and modern developments. Anaesthesia. (2019) 74:373–9. doi: 10.1111/anae.14569, 30648259

[29] Bui M Nijmeijer WS Hegeman JH Witteveen A Groothuis-Oudshoorn CGM. Systematic review and meta-analysis of preoperative predictors for early mortality following hip fracture surgery. Osteoporos Int. (2024) 35:561–74. doi: 10.1007/s00198-023-06942-0, 37996546 PMC10957669

[30] Wang PW Yao XD Zhuang HF Li YZ Xu H Lin JK . Mortality and related risk factors of fragile hip fracture. Orthop Surg. (2022) 14:2462–9. doi: 10.1111/os.13417, 36017769 PMC9531092

[31] Lu Q Chen M Ling H. Prediction of 1-year post-operative mortality in elderly patients with fragility hip fractures in China: evaluation of risk prediction models. Front Surg. (2025) 12:1415680. doi: 10.3389/fsurg.2025.1415680, 40625675 PMC12230039

