# Supplementary material for: Application of deep learning-clinical baseline feature fusion model to predict postoperative mortality in elderly patients with hip fracture: a multicenter study
Source: Front Med (Lausanne). 2026 Apr 7;13:1784156. doi: 10.3389/fmed.2026.1784156 (PMC13095813; doi:10.3389/fmed.2026.1784156)
Supplement: Supplementary file 1 [file Supplementary_file_1.docx]

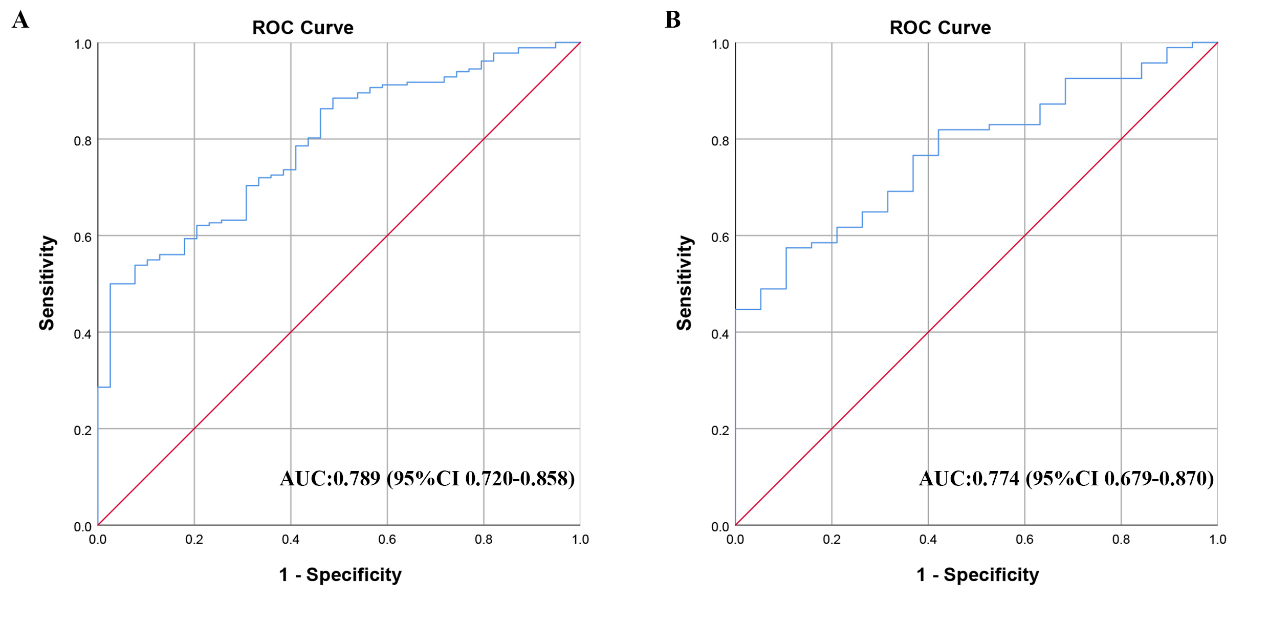


**Figure S1,** Receiver operating characteristic curves of clinical prediction model in A) training and B) validation sets.


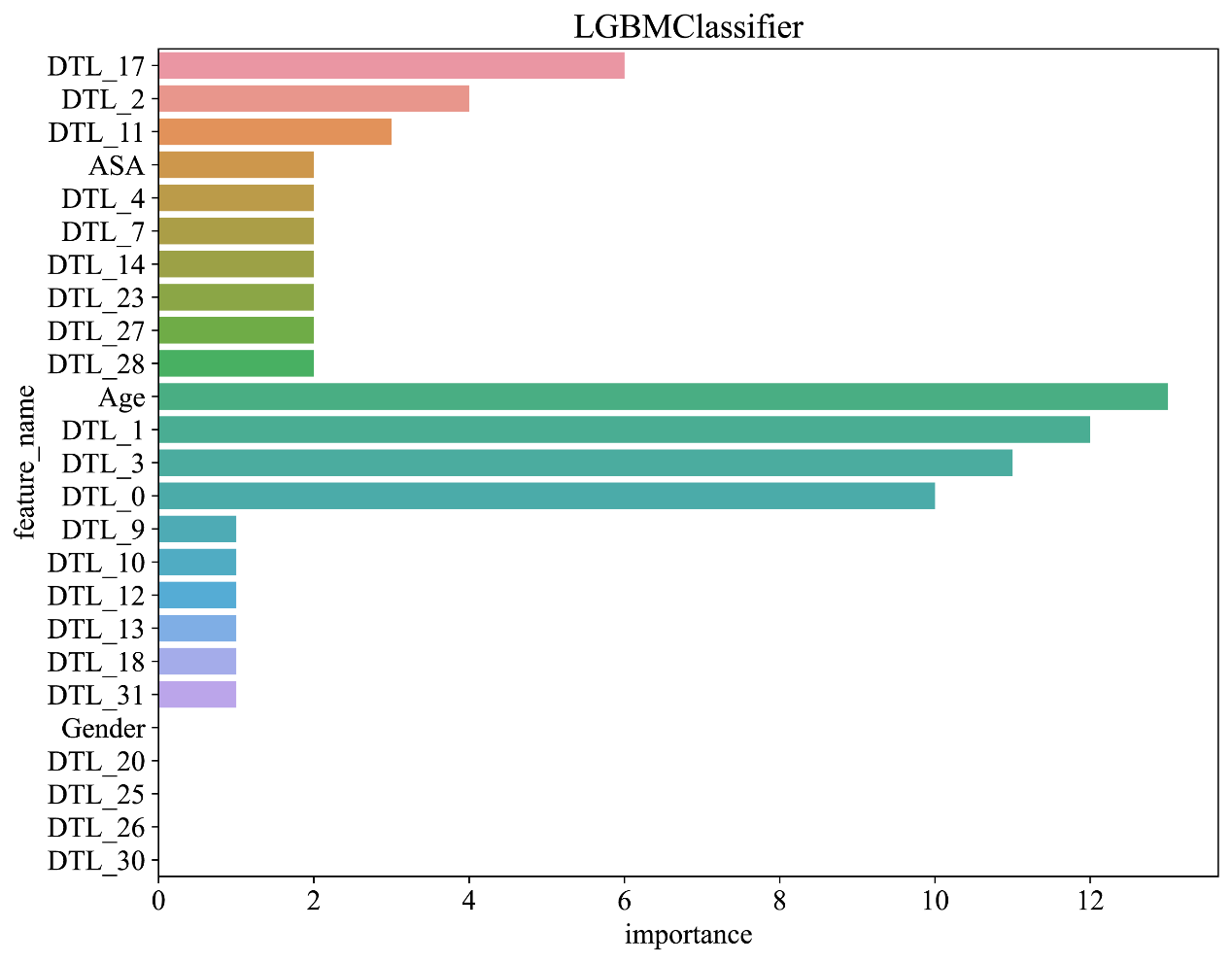


**Figure S2**, The importance ratio of different features in the LightGBM fusion model.

**Abbreviations** DTL, Deep Learning Tissue Feature; ASA, American Society of Anesthesiologists physical status score


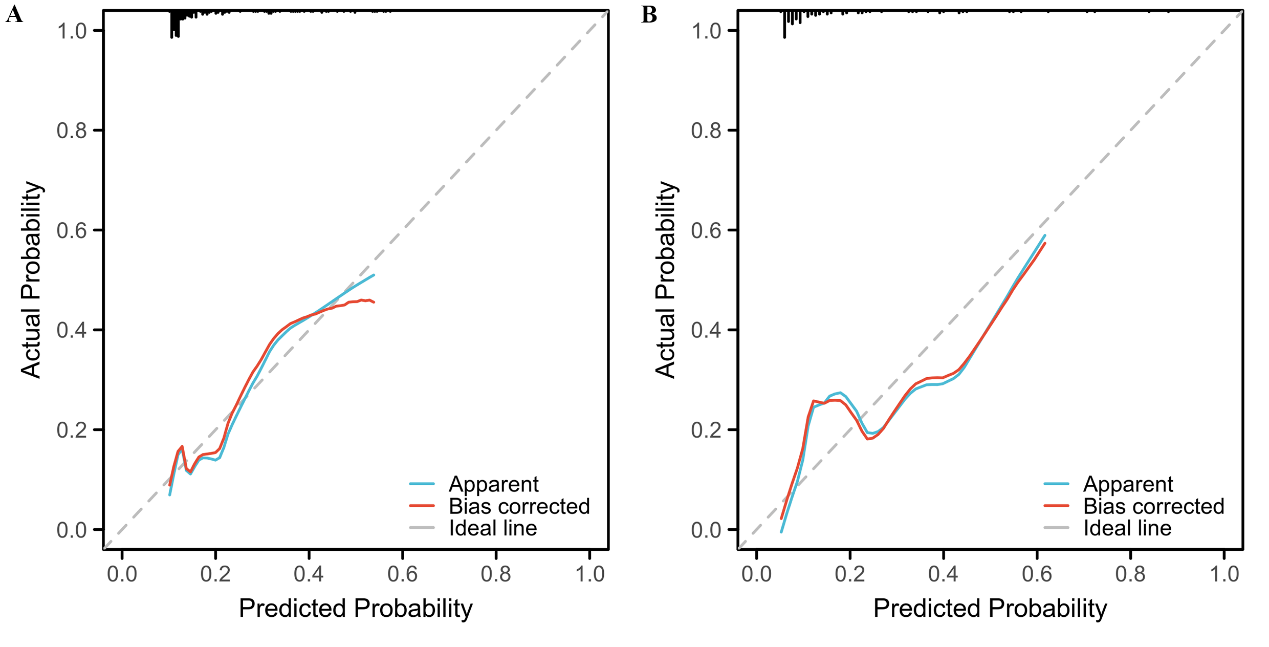


**Figure S3,** Calibration curves of the LightGBM fusion model: A) training and B) validation sets.
